# Two New Acylated Flavonol Glycosides from the Seeds of *Lepidium sativum*

**DOI:** 10.3390/molecules190811341

**Published:** 2014-07-31

**Authors:** Qing-Lu Fan, Yin-Di Zhu, Wen-Hua Huang, Yun Qi, Bao-Lin Guo

**Affiliations:** Key Laboratory of Bioactive Substances and Resources Utilization of Chinese Herbal Medicine, Institute of Medicinal Plant Development, Chinese Academy of Medical Sciences and Peking Union Medical College, Beijing 100193, China; E-Mails: fanqinglu2007@126.com (Q.-L.F.); zhuyindi314@sina.com (Y.-D.Z.); hwhzh69@sohu.com (W.-H.H.); yunqicha@sohu.com (Y.Q.)

**Keywords:** *Lepidium sativum*, flavonol glycoside, anti-inflammatory activity

## Abstract

Two new acylated flavonol glycosides named kaempferol-3-*O*-(2-*O*-sinapoyl)-β-d-galactopyranosyl-(1→2)-β-d-glucopyranoside-7-*O*-α-l-rhamnopyranoside (**1**) and quercetin-3-*O*-(6-*O*-benzoyl)-β-d-glucopyranosyl-(1→3)-β-d-galactopyranoside-7-*O*-α-l-rhamnopyranoside (**2**), were isolated together with six known compounds from the seeds of *L. sativum*. Their structures were elucidated on the basis of spectroscopic analysis and chemical methods. *In vitro* **1** and **2** inhibited nitric oxide production in lipopolysaccharide (LPS)-stimulated RAW 264.7 cells, with IC_50_ values of 25.36 and 25.08 µM, respectively.

## 1. Introduction

*Lepidium sativum* L. (Cruciferae), also known as “garden cress”, is a fast-growing annual herb popularly used for its wide therapeutic application including anti-inflammatory [1], hypoglycemic [2], antihypertensive [3], fracture healing activities [4] and efficacy in gastrointestinal diseases [5]. In some regions, seedlings of *L. sativum* are also used in salads because of their pungent taste. Previous phytochemical investigations disclosed the presence of sinapic acid, alkaloids [6], flavonoids [7], steryl ester [8] and terpenes [9]. In the course of our search for novel bioactive agents, two new acylated flavonol glycosides, kaempferol-3-*O*-(2-*O*-sinapoyl)-β-d-galactopyranosyl-(1→2)-β-d-glucopyranoside-7-*O*-α-l-rhamnopyranoside (**1**) and quercetin-3-*O*-(6-*O*-benzoyl)-β-d-glucopyranosyl-(1→3)-β-d-galacto-pyranoside-7-*O*-α-l-rhamnopyranoside (**2**), were isolated from the seeds of *L. sativum* along with six known compounds. Herein, we report the isolation and characterization of new compounds, as well as their inhibitory activities against NO production induced by LPS and α-glucosidase.

## 2. Results and Discussion

**Figure 1:**
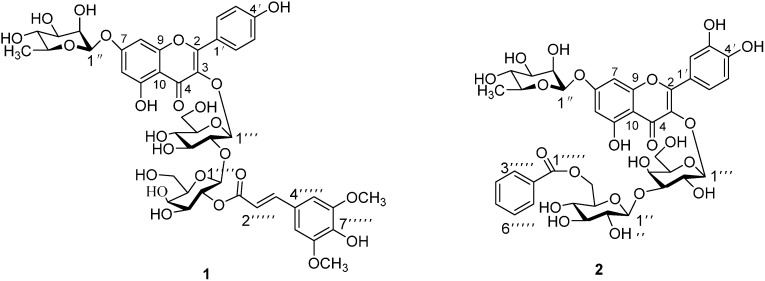
Chemical structures of **1** and **2**.

Compound **1** was isolated as a yellow amorphous powder. Its UV spectrum exhibited a characteristic flavonol absorption band at 268 nm. The HRESIMS spectrum showed a quasi-molecular ion at *m/z* 985.2630 (calc. for C_44_H_50_O_24_Na, 985.2589), from which in conjunction with NMR data the molecular formula was established as C_44_H_50_O_24_, suggesting twenty indices of hydrogen deficiency. The ^1^H-NMR spectrum (Table 1) indicated the presence of kaempferol as an aglycone [*δ*_H_ 6.32 (1H, d, *J* = 2.4 Hz, H-6), 6.63 (1H, d, *J* = 2.4 Hz, H-8), 6.89 (2H, d, *J* = 9.0 Hz, H-3ꞌ, 5ꞌ), 8.01 (2H, d, *J* = 9.0 Hz, H-2ꞌ, 6ꞌ)], two methoxyl groups at *δ*_H_ 3.72 (6H, s), two benzene protons at *δ*_H_ 6.70 (2H, s), and *trans*-olefinic protons at *δ*_H_ 6.29 and *δ*_H_ 7.37 (each d, *J* = 16.2 Hz), and three anomeric protons at *δ*_H_ 5.50, 5.81, 5.08 . Except for carbon signals of an kaempferol, The ^13^C-NMR spectrum showed 29 carbon signals, including one carbonyl (*δ*_C_ 165.7), six benzene carbons [*δ*_C_ 105.4 (×2), 124.3, 137.9, 147.7 (×2)], two olefinic carbons (*δ*_C_ 115.2, 144.5), and two methoxy groups [*δ*_C_ 55.8 (×2)] ascribed to a sinapoyl group, three anomeric carbon signals at *δ*_C_ 97.0, 98.3, 98.5. NMR data indicated that **1** is an acylated kaempferol glycoside. All the ^1^H- and ^13^C-NMR data of **1** were assigned by TOCSY, HSQC, and HMBC experiments. Acid hydrolysis of **1** afforded kaempferol, glucose, rhamnose and galactose, identified by direct TLC comparison with authentic samples. The absolute configurations of the sugars were determined by GC analysis to be d- for glucose and galactose, and l- for rhamnose. Unequivocal assignment could be achieved by 2D-NMR spectra. The HMBC spectrum showed correlations between *δ*_H_ 5.50 (Rha H-1) and 161.50 (C-7), 5.81(H-1_glc_) and 133.13 (C-3), 5.08 (H-1_gal_) and 77.42 (C-2_glc_), 3.77 (H-2_glc_) and 98.28 (C-1_gal_), 4.69 (H-2_gal_) and 165.66 (C-9_sinapoyl_) (Figure 1). Detailed analyses of the ^1^H- (δ 5.81, d, *J* = 7.8 Hz, H-1''') and ^13^C-NMR (δ 97.0, 77.4, 75.6, 68.1, 72.0, 61.0) suggested glucopyranose as the sugar moiety, the C-2 (glu) was shifted downfield at δ 77.4, indicating that glycosylation of the galactose unit by the glucopyranosyl was on the 2-hydroxyl. A downfield shift of C-2'''' was from δ C 71.2 to 73.5, and an upfield shift of C-1'''' was from δ C 103.4 to 98.3, which were in accordance with the acylation of C-2ꞌꞌꞌꞌ of the galactose moiety [10]. Moreover, the downfield shift of H-2ꞌꞌꞌꞌ to 4.69 (dd, *J* = 9.6, 9.0 Hz) further confirmed the presence of a C-2ꞌꞌꞌꞌ sinapoyl in compound **1** [11]. The β configuration of the anomeric carbon of glucose and aglycone were inferred from the coupling constant of H-1ꞌꞌꞌ (*J* = 7.8 Hz) observed in the ^1^H-NMR spectrum [12]. The coupling constant of H-1ꞌꞌꞌꞌ (*J* = 7.8 Hz) demonstrated that the galactose was in the β-orientation, while the smaller coupling constant value (*J* = 1.2 Hz) indicated that the rhamnosyl group was α-linked to the aglycone. Thus, **1** was a new compound identified as kaempferol-3-*O*-(2-*O*-sinapoyl)-β-d-galactopyranosyl-(1→2)-β-d-glucopyranoside-7-*O*-α-l-rhamnopyranoside.

**Table 1:** ^1^H and ^13^C-NMR data for compounds **1** and **2** (600 and 150 MHz, DMSO-*d*_6_, δ ppm).

No.	1			2		
	δC	δH (mult, *J* in Hz)	HMBC	δC	δH (mult, *J* in Hz)	HMBC
2	155.7			155.7		
3	133.1			133.3		
4	177.4			177.4		
5	160.7	OH (12.66)		160.9	12.65 (OH)	
6	99.3	6.32(d, 2.4)	C-5, C-7, C-8	99.2	6.38 (d, 1.8)	C-5, 8, 10
7	161.5			161.4		
8	93.8	6.63(d, 2.4)	C-7, C-9, C-10	94.1	6.69 (d, 1.8)	C-6, 7, 9, 10
9	155.7			155.6		
10	105.5			105.5		
1ꞌ	120.9			122.2		
2ꞌ	130.9	8.01 (d, 9)	C-2, C-4ꞌ	115.8	7.50 (d, 1.8)	C-1ꞌ, 3ꞌ, 4ꞌ, 2
3ꞌ	115.4	6.89 (d, 9 )	C-4ꞌ	144.9		
4ꞌ	156.0	OH (s, 10.18)		148.8		
5ꞌ	115.4	6.89 (d, 9)	C-4ꞌ	115.2	6.82 (d, 8.4)	C-3ꞌ, 4ꞌ, 6ꞌ
6ꞌ	130.9	8.01 (d, 9)	C-2ꞌ	120.7	7.65 (dd, 8.4, 1.8)	C-2ꞌ, 4ꞌ, 5ꞌ, 2
1ꞌꞌ	98.5	5.50 (d, 1.2)	C-7, 3ꞌꞌ	98.4	5.54 (s)	C-7, 2ꞌꞌ
2ꞌꞌ	69.8	3.87 (br. m)	C-3ꞌꞌ, 4ꞌꞌ	69.8	3.86 (br. s)	C-5ꞌꞌ
3ꞌꞌ	70.2	3.63 (m)		70.3	3.65 (dd, 3.0, 9.0 )	
4ꞌꞌ	71.6	3.31 (m)	C-3ꞌꞌ	71.6	3.33 (m)	C-2ꞌꞌ, 6ꞌꞌ
5ꞌꞌ	70.0	3.43 (m)	C-4ꞌꞌ, 6ꞌꞌ	70.1	3.45 (m)	C-3ꞌꞌ, 4ꞌꞌ
6ꞌꞌ	18.0	1.13 (3H, d, 6.0)	C-4ꞌꞌ, 5ꞌꞌ	17.9	1.14 (d, 6.0)	C-4ꞌꞌ, 5ꞌꞌ
1ꞌꞌꞌ	97.0	5.81 (d, *J* = 7.8)	C-3, 2ꞌꞌꞌ, 5ꞌꞌꞌ	98.5	5.59 (d, 7.8 )	C-3
2ꞌꞌꞌ	77.4	3.77 (dd, 9.6, 7.8)	C-5ꞌꞌꞌ, 1ꞌꞌꞌ	72.9	3.61 (dd, 9.0, 7.8)	C-3ꞌꞌꞌ
3ꞌꞌꞌ	75.6	3.34 (m)	C-2ꞌꞌꞌ	81.9	3.76 (dd, 9.0, 4.0)	C-1ꞌꞌꞌꞌ, C-1ꞌꞌꞌ, C-2ꞌꞌꞌ
4ꞌꞌꞌ	68.1	3.65 (m)	C-5ꞌꞌꞌ	67.5	3.68 (dd, 4.0, 3.0)	C-ꞌꞌꞌ, 3ꞌꞌꞌ
5ꞌꞌꞌ	72.0	3.62 (m)	C-6ꞌꞌꞌ	75.8	3.30 (m)	C-1ꞌꞌꞌ, 4ꞌꞌꞌ, 6ꞌꞌꞌ
6ꞌꞌꞌ	61.0	3.72 (m) 3.51 (m)		59.9	3.23 (m), 3.38 (m)	C-4ꞌꞌꞌ, 5ꞌꞌꞌ
1ꞌꞌꞌꞌ	98.3	5.08 (d, 7.8)	C-2ꞌꞌꞌ	105.0	4.59 (d, 7.8 )	C-3ꞌꞌꞌ, 2ꞌꞌꞌꞌ, 5ꞌꞌꞌꞌ
2ꞌꞌꞌꞌ	73.5	4.69 (dd, 9.0, 7.8)	C-1ꞌꞌꞌꞌ, 3ꞌꞌꞌꞌ, 1ꞌꞌꞌꞌꞌ	74.8	3.15 (dd, 9.0, 7.8 )	C-1ꞌꞌꞌꞌ, 5ꞌꞌꞌꞌ
3ꞌꞌꞌꞌ	74.4	3.45 (m)	C-2ꞌꞌꞌꞌ, 4ꞌꞌꞌꞌ	74.0	3.56 (dd, 9.0, 7.2)	C-4ꞌꞌꞌꞌ
4ꞌꞌꞌꞌ	70.4	3.23 (m)	C-3ꞌꞌꞌꞌ, 5ꞌꞌꞌꞌ	70.0	3.23 (m)	C-5ꞌꞌꞌꞌ
5ꞌꞌꞌꞌ	76.6	3.28 (m)	C-4ꞌꞌꞌꞌ	76.0	3.30	C-4ꞌꞌꞌꞌ
6ꞌꞌꞌꞌ	59.9	3.23 (m) 3.36 (m)	C-3ꞌꞌꞌꞌ, 5ꞌꞌꞌꞌ	64.2	4.30 (dd, 6.0, 12.0) 4.40 (br. d, 12.0)	C-1ꞌꞌꞌꞌꞌ, C-3ꞌꞌꞌꞌ C-1ꞌꞌꞌꞌꞌ, 3ꞌꞌꞌꞌ
1ꞌꞌꞌꞌꞌ	165.7			165.4		
2ꞌꞌꞌꞌꞌ	115.2	6.31 (d, 16.2)	C-4ꞌꞌꞌꞌꞌ, C-1ꞌꞌꞌꞌꞌ	129.4		
3ꞌꞌꞌꞌꞌ	144.5	7.37 (d, 16.2)	C-1ꞌꞌꞌꞌꞌ, 2ꞌꞌꞌꞌꞌ, 4ꞌꞌꞌꞌꞌ, 5ꞌꞌꞌꞌꞌ, 9ꞌꞌꞌꞌꞌ	128.7	7.69 (d, 7.2)	C-1ꞌꞌꞌꞌꞌ, 5ꞌꞌꞌꞌꞌ
4ꞌꞌꞌꞌꞌ	124.3			128.1	7.20 (t, 7.2)	C-1ꞌꞌꞌꞌꞌ, 3ꞌꞌꞌꞌꞌ, 7ꞌꞌꞌꞌꞌ, 5ꞌꞌꞌꞌꞌ
5ꞌꞌꞌꞌꞌ	105.4	6.70 (s)	C-3ꞌꞌꞌꞌꞌ, 4ꞌꞌꞌꞌꞌ 6ꞌꞌꞌꞌꞌ, 7ꞌꞌꞌꞌꞌ, 8ꞌꞌꞌꞌꞌ	132.7	7.38 (t, 7.2)	C-3ꞌꞌꞌꞌꞌ, 7ꞌꞌꞌꞌꞌ
6ꞌꞌꞌꞌꞌ	147.7			128.1	7.20 (t, 7.2 )	C-1ꞌꞌꞌꞌꞌ, 3ꞌꞌꞌꞌꞌ, 7ꞌꞌꞌꞌꞌ, 5ꞌꞌꞌꞌꞌ
7ꞌꞌꞌꞌꞌ	137.9	8.75 (OH)		128.7	7.69 (d, 7.2)	C-1ꞌꞌꞌꞌꞌ, 5ꞌꞌꞌꞌꞌ
8ꞌꞌꞌꞌꞌ	147.7					
9ꞌꞌꞌꞌꞌ	105.4	6.70 (s)	C-3ꞌꞌꞌꞌꞌ, 4ꞌꞌꞌꞌꞌ, 6ꞌꞌꞌꞌꞌ, 7ꞌꞌꞌꞌꞌ, 8ꞌꞌꞌꞌꞌ			
CH_3_O	55.8	3.72 (s) × 2	C-5ꞌꞌꞌꞌꞌ, 6ꞌꞌꞌꞌꞌ, 8ꞌꞌꞌꞌꞌ, 9ꞌꞌꞌꞌꞌ			

Compound **2** was also obtained as a yellow amorphous powder with a molecular formula C_40_H_44_O_22_ ([M+Na]^+^ *m/z* 899.2222) on HRESIMS. The UV spectrum was similar to that of **1**, suggesting a flavonol glycoside structure. Acid hydrolysis and GC analysis showed that the sugar units of **2** were the same as **1**. Comparison of NMR spectroscopic data of **2** (Table 1) with those of **1** indicated that the difference of both compounds were the aglycone and acyl moiety, the kaempferol and sinapoyl group in **1** being replaced by quercetin and benzoyl in **2**, respectively. The higher frequency signals in ^1^H-NMR at 7.69 (2H, d, *J* = 7.2 Hz), 7.20 (2H, t, *J* = 7.2 Hz) and 7.38 (t, *J* = 7.2 Hz) indicated the presence of a benzoyl moiety in **2**. The higher frequency chemical shift of hydroxymethylene (C-6ꞌꞌꞌꞌ at δ 64.2 revealed the attachment of a benzoyl moiety at glucose. Detailed comparison of the ^13^C-NMR and HMBC spectra between the two compounds indicated that the difference was in the link location and link order of the glycoside moiety. The carbon signals at δC 98.5 (C-1ꞌꞌꞌ), 72.9 (C-2ꞌꞌꞌ), 81.9 (C-3ꞌꞌꞌ), 67.5 (C-4ꞌꞌꞌ), 75.8 (C-5ꞌꞌꞌ) and 59.9 (C-6ꞌꞌꞌ), together with an HMBC correlation between C-3 and H-1ꞌꞌꞌ (5.59, *J* = 7.8 Hz) revealed a galactopyranoside moiety was located on C-3 [13]. The HMBC experiment indicated correlations between *δ*_H_ 5.54 (H-1_rha_) and *δ*_C_ 161.38 (C-7), *δ*_H_ 5.60 (H-1_gal_) and *δ*_C_ 133.26 (C-3), *δ*_H_ 4.60 (H-1_glc_) and *δ*_C_ 81.94 (C-3_gal_), *δ*_H_ 3.76 (H-3_gal_) and *δ*_C_ 105.03 (C-1_glc_), *δ*_H_ 4.30 (H-6_glc_) and *δ*_H_ 165.40 (C-1_benzoyl_) (Figure 1). Consequently, **2** was a new compound and identified as quercetin-3-*O*-(6-*O*-benzoyl)-β-d-glucopyranosyl-(1→3)-β-d-galactopyranoside-7-*O*-α-l-rhamnopyranoside.

The known compounds were identified as isorhamnetin (**3**), quercetin (**4**), kaempferol (**5**), osthole (**6**), protocatechuic acid (**7**), and staphylionosides A (**8**), respectively, on the basis of their spectroscopic data and by comparison of their spectroscopic data with previously published values [14,15,16,17,18,19].

Considering the medical applications of *L. sativum*, **1** and **2** were evaluated for their inhibitory effects on NO release in the lipopolysaccharide stimulated RAW 264.7 macrophage cell line and their α-glucosidase inhibitory activity. The results showed that compounds **1** and **2** inhibited NO production in LPS-stimulated RAW 264.7 cells with IC_50_ values of 24.40 µg/mL and 21.97 µg/mL, respectively. Compounds **1** and **2** also exhibited α-glucosidase inhibitory activity at 20 µg/mL and the inhibitory activity was 10.50 and 8.93, respectively. The inhibitory rate (%) of compound **1** at 6.50 µM (6.25 µg/mL), 13.00 µM (12.5 µg/mL), 26.00 µM (25 µg/mL) and 52.00 µM (50 µg/mL) were 20.82, 31.67, 43.99 and 96.77, respectively. The inhibitory rate (%) of compound **2** at 7.13 µM (6.25 µg/mL), 14.27 µM (12.5 µg/mL), 28.54 µM (25 µg/mL) and 56.97 µM (50 µg/mL) were 24.93, 31.96, 67.74 and 84.46, respectively. Dexamethasone (1.27 µM) showed 27.1% inhibition [20]. Compound **1** and **2** also exhibited α-glucosidase inhibitory activity at 20.79 µM (20 µg/mL) and 22.78 µM (20 µg/mL) and the inhibitory rate (%) was 10.50 and 8.93, respectively, while the IC_50_ of acarbose was 200 μg/mL [21].

## 3. Experimental

### 3.1. General Procedures

Optical rotations were obtained on a Perkin-Elmer 341 digital polarimeter (Waltham, MA, USA). UV spectra were recorded on Shimadzu UV2550 (Tokyo, Japan). NMR spectra were obtained with a Bruker AV β 600 NMR spectrometer (chemical shift values are presented as δ values with TMS as the internal standard; Munich, Germany). HR-ESI-MS spectra were performed on a LTQ-Orbitrap XL spectrometer. GC analysis was carried out on a GC-7890: column, DB-5 (30 m × 0.32 mm × 0.25 mm); detector, FID-6850 (Agilent, Santa Clara, CA, USA). ODS gel (50 µm, YMC, Kyoto, Japan), Sephadex LH-20 (Pharmacia, Uppsala, Sweden), and MDS gel (Beijing Medicine Technology Center, Beijing, China) were used for column chromatography. HPLC separations were performed using a Waters 2535 series pump equipped with a PDA detector and a YMC (250 × 10 mm, 5 μm) semi-preparative column. TLC was carried out on silica gel GF_254_ (Yantai Chemical Inst., Yantai, China) plates, and spots were visualized under UV light (254 or 365 nm) or by spraying with 5% H_2_SO_4_ in 95% EtOH followed by heating.

### 3.2. Plant Material

The seeds of *L. sativum* were purchased from the Xinjiang Uygur Autonomous Region in August 2010. The plant material was authenticated by one of the authors (B.-L. Guo). A voucher specimen is deposited at the Natural Medicine Research Center of Institute of Medicinal Plant Development, Chinese Academy of Medical Sciences and Peking Union Medical College.

### 3.3. Extraction and Isolation

The dried seeds of *L. sativum* (45 kg) were chopped and extracted with 95% EtOH (270 L) three times (each time 8 h) under percolation and then concentrated under vacuum. The residue was extracted three times under reflux by 50% EtOH for 1.5 h. The dried 95% EtOH extract was further suspended in water and partitioned successively with petroleum ether, CHCl_3_, EtOAc and *n*-BuOH (50 L, 80 L, 100 L, 100 L). After concentration the *n*-BuOH layer (400 g) was subjected to column chromatography on MDS gel column (15 cm × 47 cm, 75–150 μm) eluted with a gradient of MeOH–H_2_O (5:95, 10:90, 15:85, 20:80, 30:70, 50:50, 70:30, 100:0, *v/v*) to give seven factions (Fr.1–Fr.7) according to TLC analyses. Fr.6 (MeOH–H_2_O 1:1, *v/v*) was subjected to chromatography on a ODS gel (5 cm × 50 cm, 50 μm) column with gradient elution (MeOH–H_2_O, 10:90, 20:80, 30:70, 40:60, 50:50, 70:30, 100:0, *v/v*), to give seven subfractions (Fr.6a–Fr.6g). From Fr.6c, compound **1** (343 mg) was obtained by repeated Sephadex LH-20 (MeOH–H_2_O, 1:1, *v/v*) chromatography. Compounds **2** (25 mg) and **3** (5 mg) was obtained by semi-preparative HPLC (MeOH–H_2_O, 2:3, *v/v*) from Fr.6d. Fr.5 (MeOH–H_2_O 3:7) was subjected to ODS gel column chromatography (3.2 cm × 50 cm, 50 μm, MeOH–H_2_O, 20:80, 30:70, 35:65, 40:60, 100:0 *v/v*) to afford five subfractions (Fr.5a–Fr.5e). From fraction Fr.5b compound **4** (13 mg) was isolated using semi-preparative HPLC (MeOH–H_2_O, 2:3). Fraction Fr.5c was further purified by semi-preparative HPLC with MeOH–H_2_O (2:3) to afford compound **5** (15 mg).

The 50% EtOH extract which was dissolved in water and chromatographed on a D101 macroporous adsorptive resin column eluting with a gradient of EtOH–H_2_O (0:100, 10:90, 20:80, 30:70, 50:50, 95:5, *v/v*), the eluates were concentrated under reduced pressure to dryness and six fractions were obtained.

The 30% ethanol eluate was subjected to MDS-gel chromatography (5 cm × 50 cm, 75–150 μm, MeOH–H_2_O, 20:80, 25:75, 30:70, 35:65, 40:60, 100:0 *v/v*), and the fourth fraction (MeOH–H_2_O, 35:65 ) was separated by ODS (5 cm × 50 cm, 50 μm, MeOH–H_2_O, 38:62, isocratic elution) and Sephadex LH-20 (MeOH ), to yield compounds **6**–**8** (15 mg, 13 mg, 5 mg, respectively).

### 3.4. Spectroscopic Data

Compound **1**, Yellow amorphous powder; 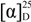 = −127.7° (*c =* 0.065, MeOH); UV λ_max_ (MeOH) nm: 224, 268, and 331 nm; IR *ν*_max_ (KBr) cm^−1^: 3259, 1660, 1595, 1520. ^1^H- and ^13^C-NMR data, see Table 1; HR-ESI-MS *m/z* 985.2630 [M+Na]^+^, (calc for C_44_H_50_O_24_Na, 985.2589).

Compound **2**, Yellow amorphous powder; 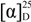 = −82.8° (*c* = 0.064, MeOH); UV (MeOH) λ_max_ 203, 257, and 358 nm; IR *ν*_max_ (KBr) cm^−1^: 3254, 1656, 1590, 1513. ^1^H- and ^13^C-NMR data, see Table 1; HR-ESI-MS *m/z* 899.2222 [M+Na]^+^, (calc. for C_40_H_44_O_22_Na: 899.2221).

### 3.5. Determination of Sugar Components

Compounds **1** (5 mg) and **2** (3 mg) were hydrolyzed with 2N TFA (5.0 mL) for 6 h in a boiling water bath. After extraction three times with CH_2_Cl_2_, the remaining aqueous layer was concentrated and identified by TLC (CHCl_3_/MeOH/H_2_O = 8:5:1) comparison with authentic samples. Spots were detected by spraying with 1% anisaldehyde (in EtOH) followed by heating. The absolute configuration of monosaccharides from each aqueous layer was determined by GC-MS of their trimethylsilylated derivatives. Column temperature: 180–250 °C, programmed increase: 15 °C/min, carrier gas: N_2_ (1 mL/min); injection temperature: 250 °C, injection volume: 1 mL. By comparing with authentic samples, l-rhamnose, d-glucose, and d-galactose were detected from **1** and **2** at t_R_: 5.815, 6.313; 7.013, and 7.713; 7.228, 7.916 min, respectively.

### 3.6. NO Inhibition Assay

Inhibition of NO production and cell viability of LPS-stimulated RAW 264.7 macrophage cells were determined. The NO production assay was carried out according to the method described before [22]. The murine monocytic RAW 264.7 macrophages were dispensed into 96-well plates (2 × 10^5^ cells/well) containing RPMI 1640 medium (Hyclone, Logan, UT, USA) with 10% FBS under humidified atmosphere of 5% CO_2_ at 37 °C. After 24 h of preincubation, cells were treated with serial dilutions of compounds **1** and **2** with the maximum concentration of 50 µM in the presence of 1 µg/mL LPS for 18 h. Each compound (purity > 95%) was dissolved in DMSO and further diluted in the medium to produce different concentrations. NO production in each well was assessed by adding 100 µL of Griess reagents A and B to 100 µL of each supernatant from LPS or the compound-treated cells in triplicate. After 5 min of incubation, the absorbance was measured at 570 nm with a 2104 Envision multilabel plate reader (Perkin-Elmer Life Sciences, Inc., Rowville, Victoria, Australia).

### 3.7. α-Glucosidase Inhibition Assay

Compounds **1** and **2** have been screened for α-glucosidase inhibitory activity with a microplate-based screening method with reference to previous literature [23]. A total of 100 μL of reaction mixture contained 25 μL of 0.1 mol/L phosphate buffer (pH 6.8), 25 μL of substratesolution (2.5 mmol/L pNPG in 0.1 mol/L phosphate buffer), 25 μL varying concentration of experimental drugs, and 25 μL of α-glucosidase solution (0.2 U/mL α-glucosidase in 0.1 mol/L phosphate buffer). After incubation of the plates at 37 °C for 15 min, 25 μL of Na_2_CO_3_ (0.2 mol/L) was added to each well to stop the reaction. The absorption was measured at 405 nm using Multiskan plate reader (Thermo Labsystems, Basingstoke, UK). The inhibitory rate (%) was calculated according to the formula: {[1 − (ODtest − ODblank)]/(control OD_test_ − control OD_blank_)} × 100%.

## 4. Conclusions

From the seeds of *L. sativum*, two new acylated flavonol glycosides, named kaempferol-3-*O*-(2-*O*-sinapoyl)-β-d-galactopyranosyl-(1→2)-β-d-glucopyranoside-7-*O*-α-l-rhamnopyranoside and quercetin-3-*O*-(6-*O*-benzoyl)-β-d-glucopyranosyl-(1→3)-β-d-galactopyranoside-7-*O*-α-l-rhamnopyranoside, were isolated. The NO production and α-glucosidase inhibitory activity were assayed *in vitro*. The two compounds showed significantly active against NO production induced by LPS. The current research demonstrates that *L. sativum* might be a great source for potential bioactive flavonol glycosides.

## Supplementary Materials

Supplementary materials can be accessed at: http://www.mdpi.com/1420-3049/19/8/11341/s1.

## Supplementary Files

Supplementary File 1
