# Supplementary material for: Two New Acylated Flavonol Glycosides from the Seeds of Lepidium sativum
Source: Molecules. 2014 Jul 31;19(8):11341–9. doi: 10.3390/molecules190811341 (PMC6271768; doi:10.3390/molecules190811341)

Supplementary Materials

Figure S1. ^{1}H-NMR spectrum of compound **1** at 600 MHz in DMSO-d_6.

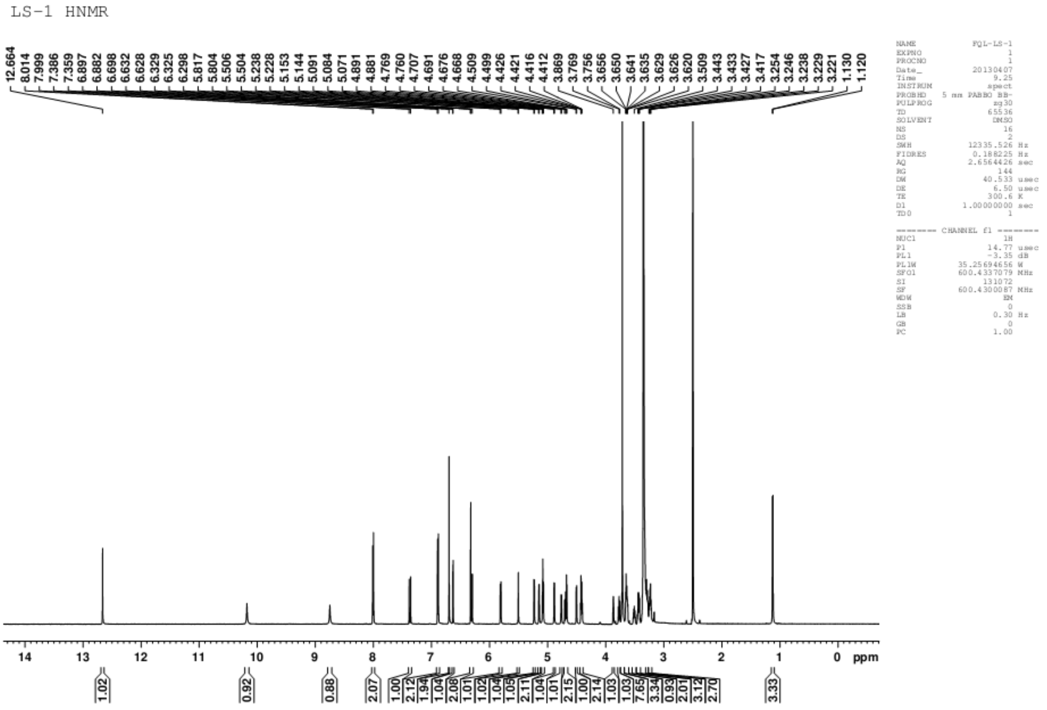

Figure S2. ^{13}C-APT spectrum of compound **1** at 150 MHz in DMSO-d_6.

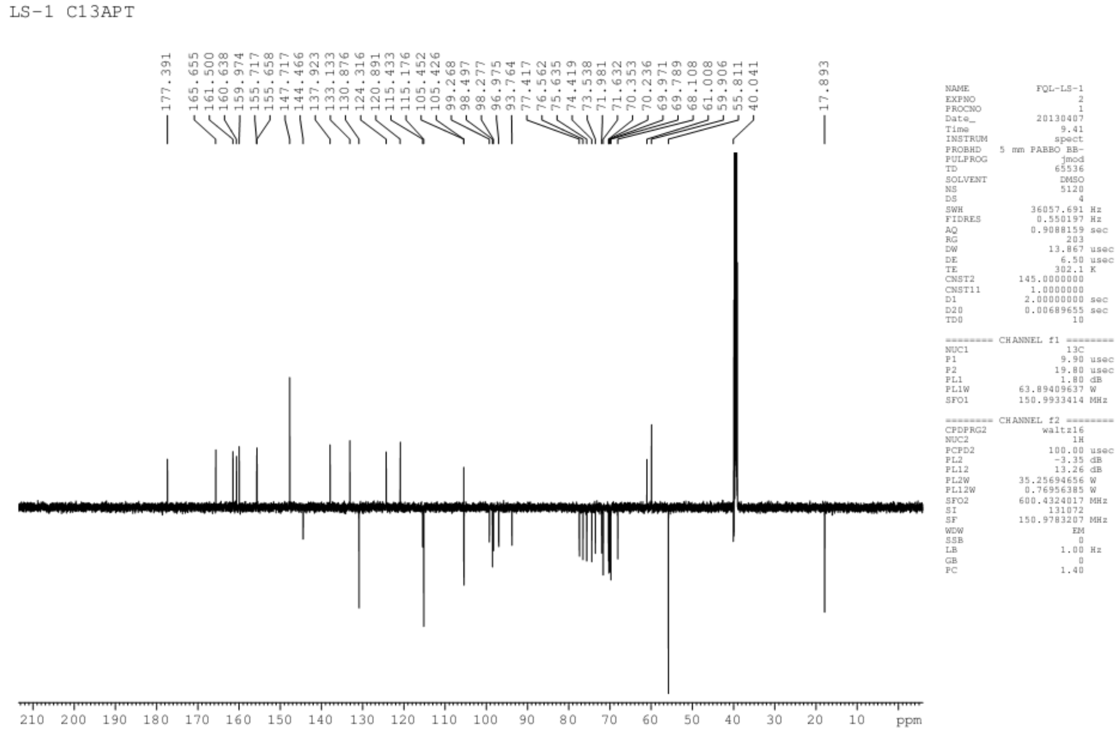

Figure S3. HSQC spectrum of compound **1** at 600 MHz in DMSO-d_6.

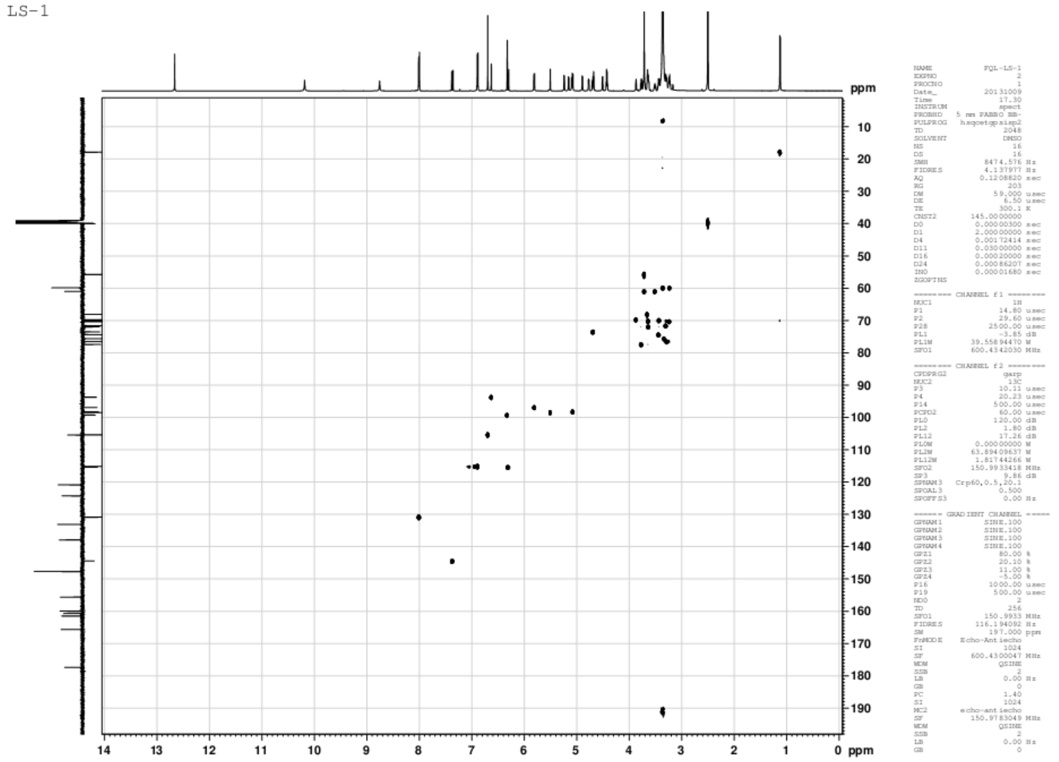

Figure S4. HMBC spectrum of compound **1** at 600 MHz in DMSO-d_6.

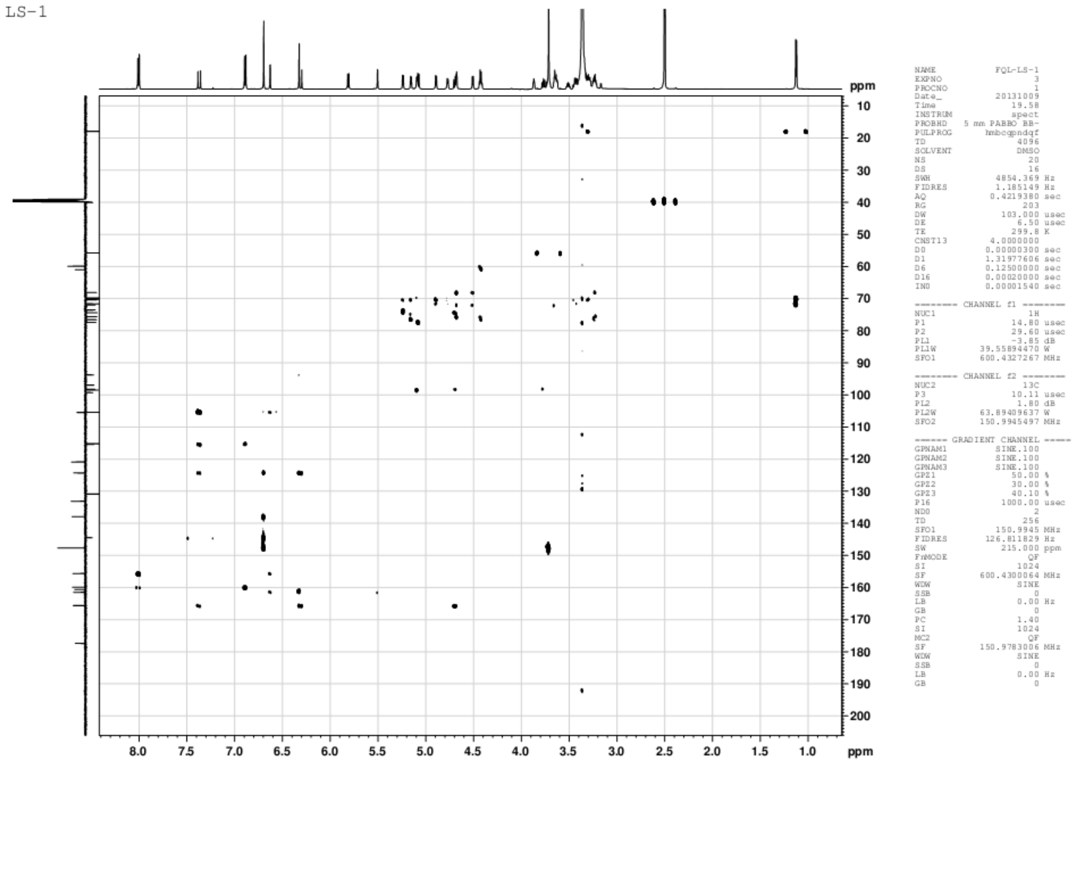

Figure S5. TOCSY spectrum of compound **1** at 600 MHz in DMSO-d_6.

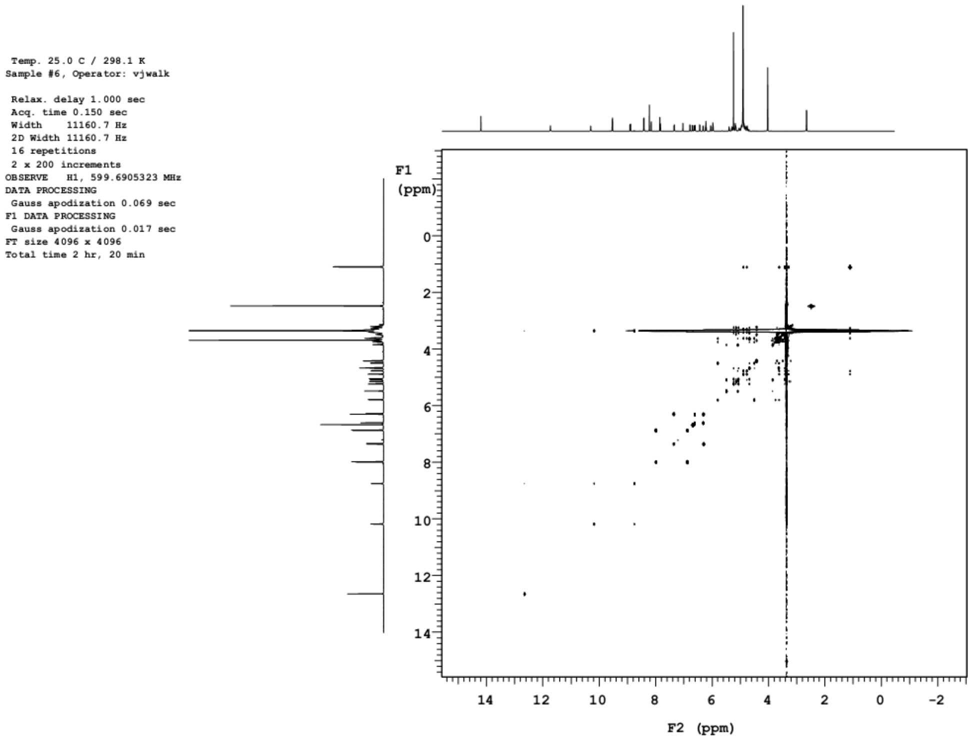

Figure S6. The HR-MS of compound **1**.

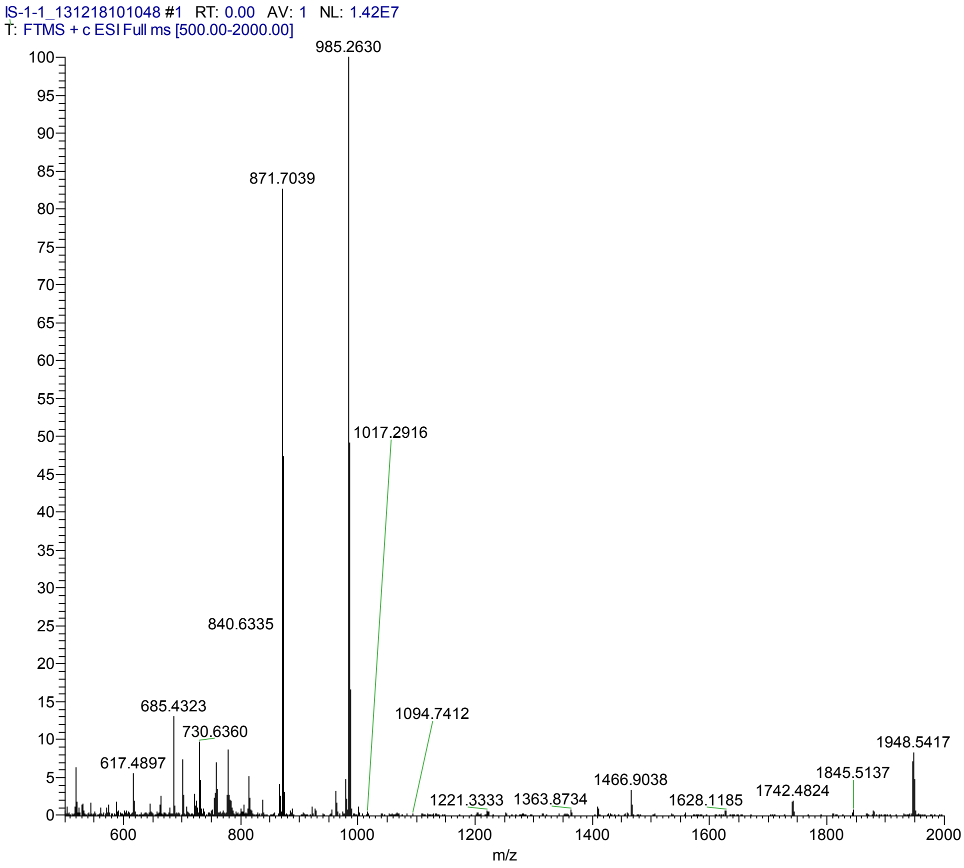

Figure S7. ^{1}H-NMR spectrum of compound **2** at 600 MHz in DMSO-d_6.

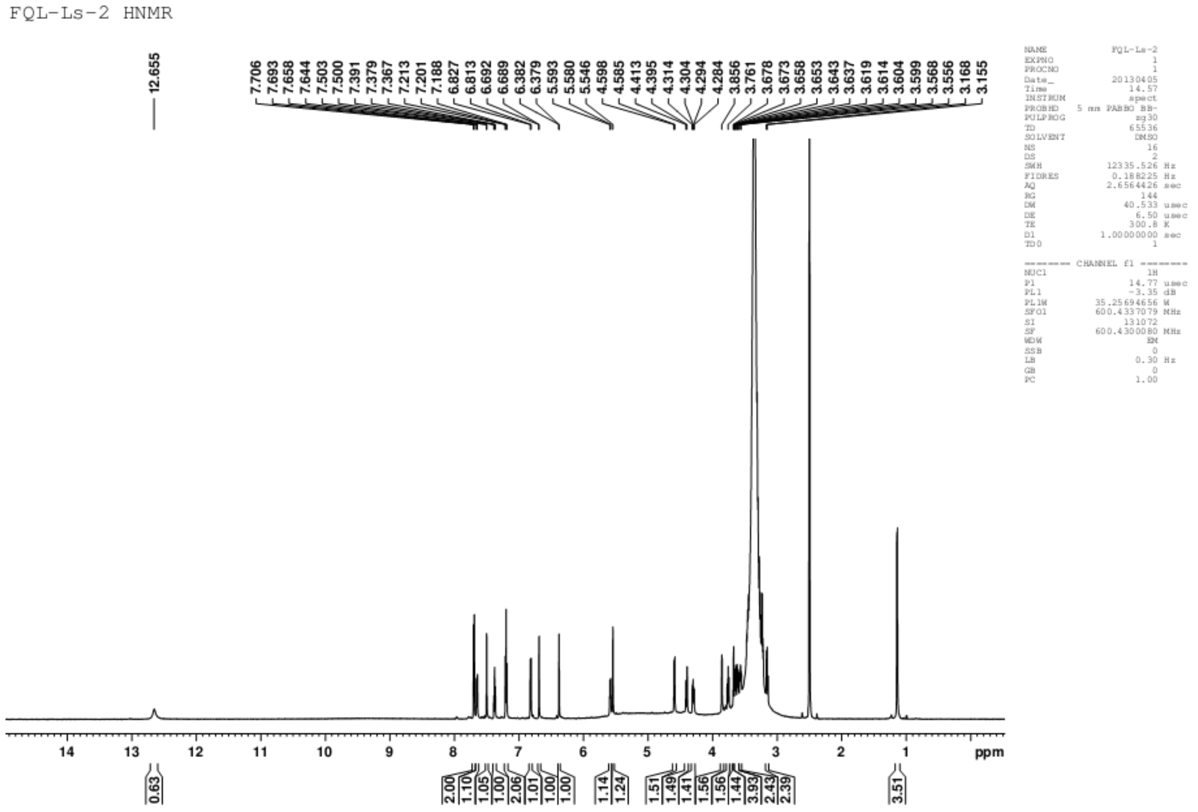

Figure S8. ^{13}C-APT spectrum of compound **2** at 150 MHz in DMSO-d_6.

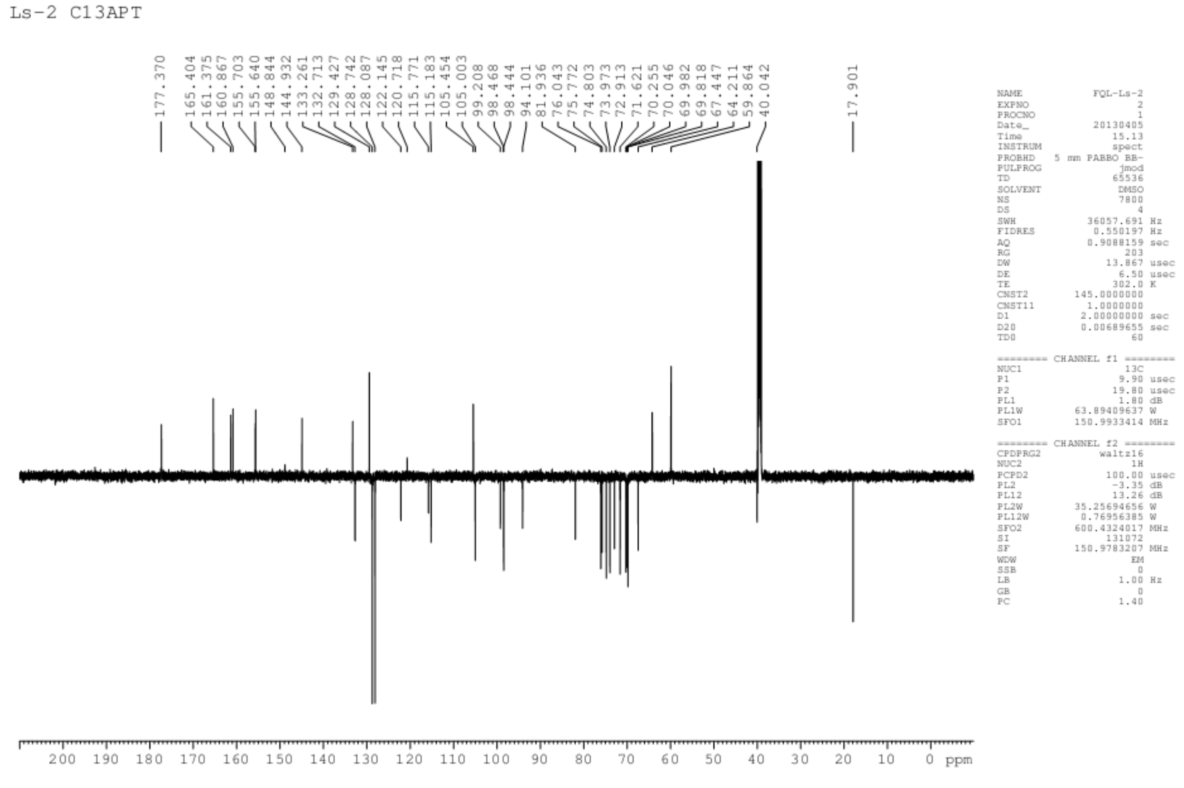

Figure S9. HSQC spectrum of compound **2** at 600 MHz in DMSO-d_6.

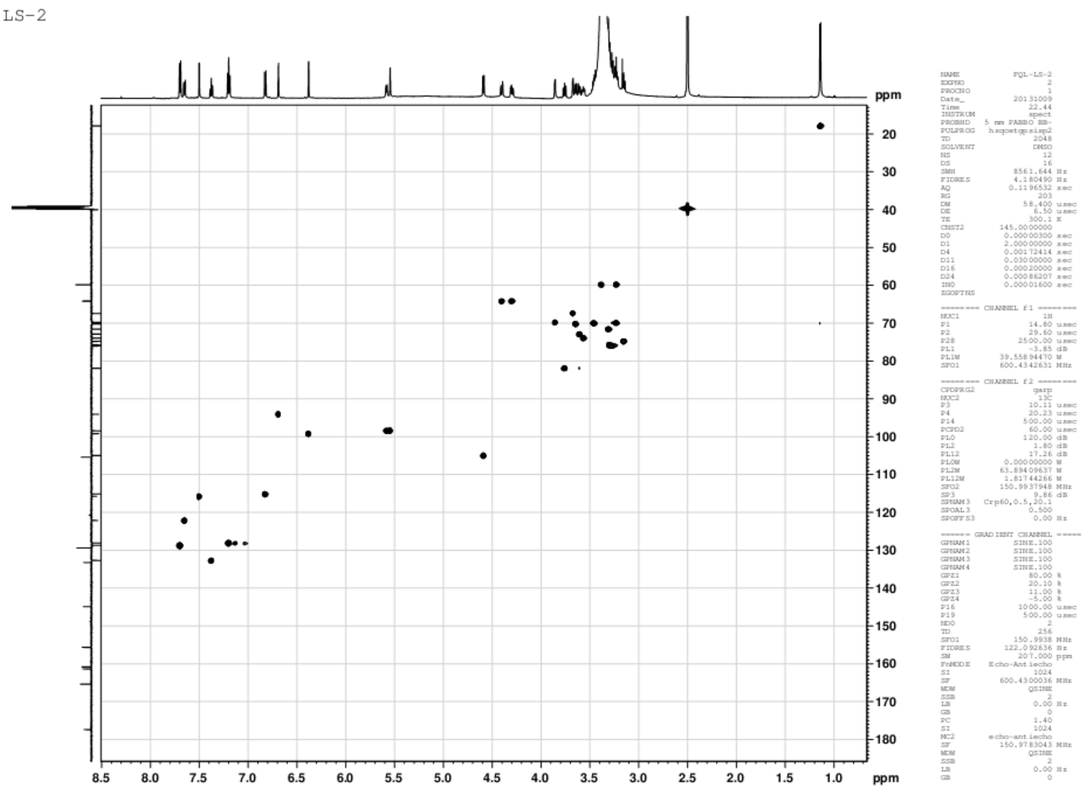

Figure S10. HMBC spectrum of compound **2** at 600 MHz in DMSO-d_6.

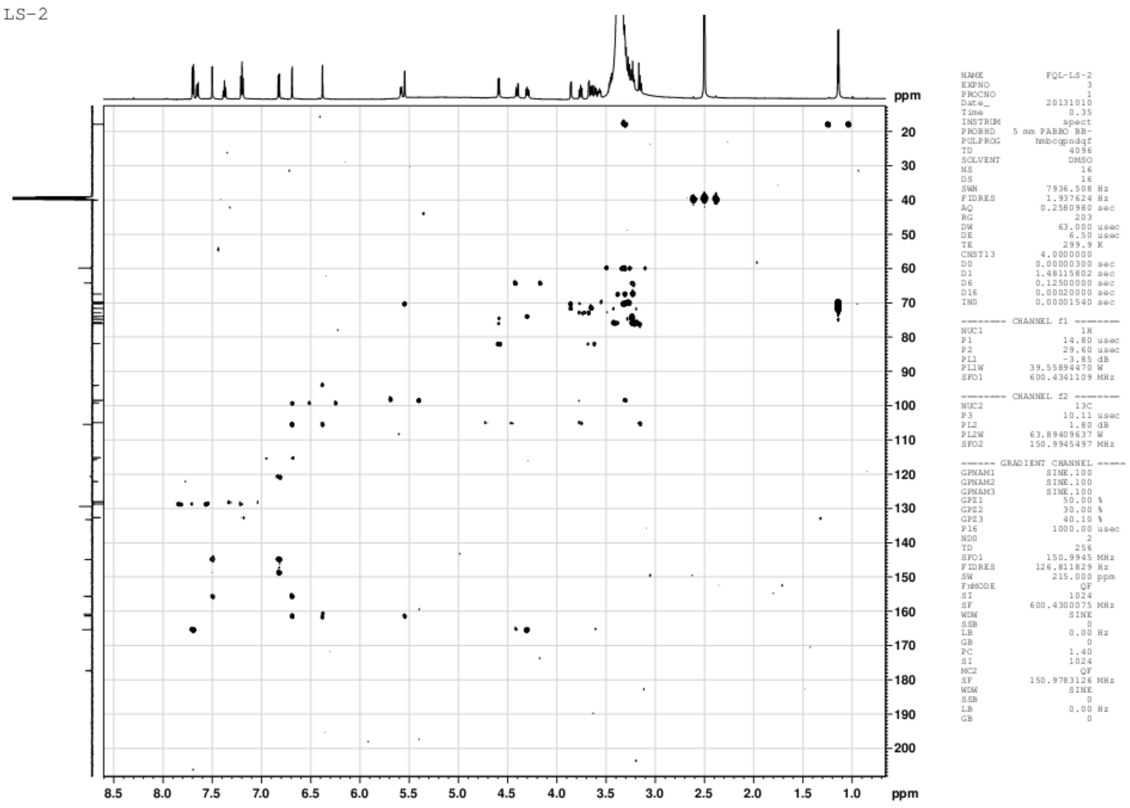

Figure S11. TOCSY spectrum of compound **2** at 600 MHz in DMSO-d_6.

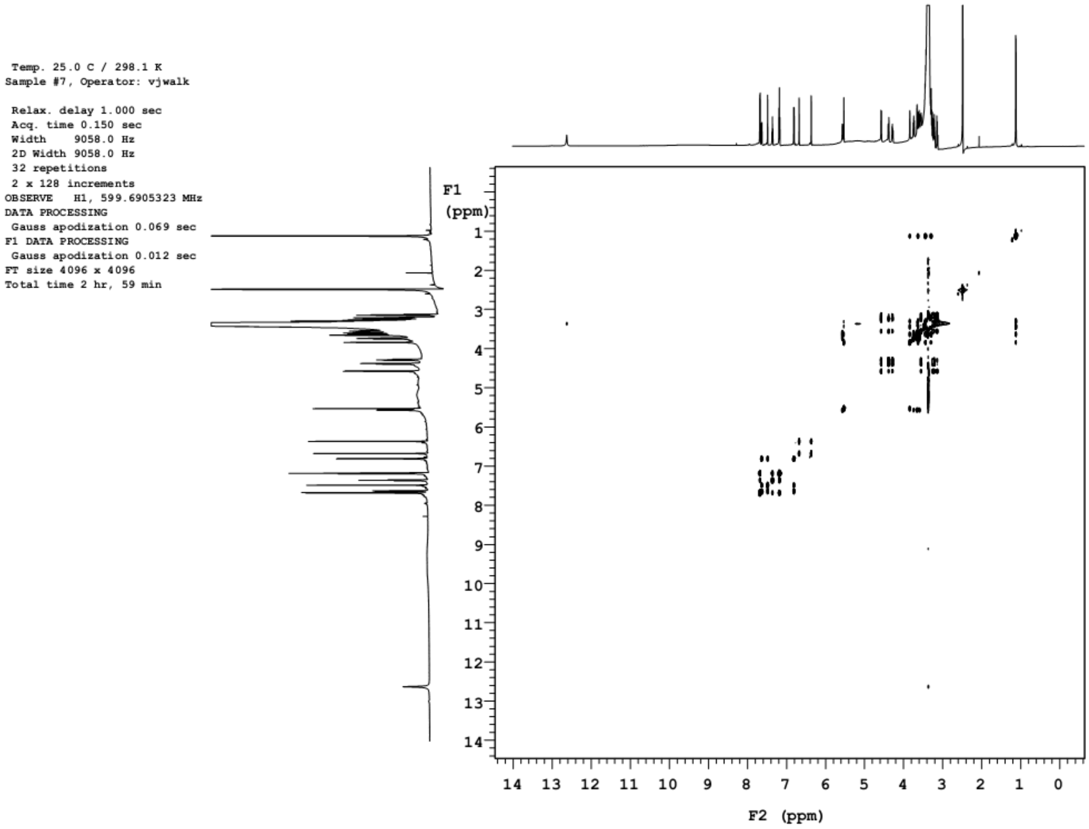

Figure S12. The HR-MS of compound **2**.

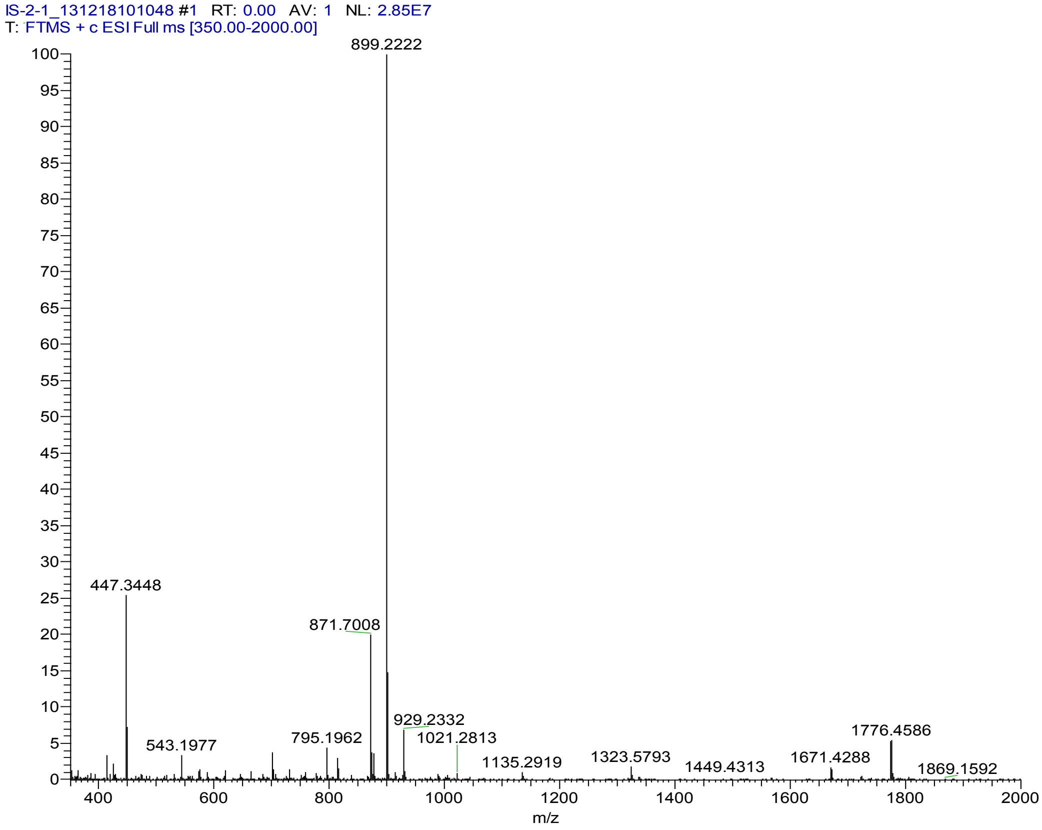

Supplement: Supplementary File 1 [file molecules-19-11341-s001.pdf]
